# Entrance Skin Dose (ESD) and Bucky Table Induced Backscattered Dose (BTI-BSD) in Abdominal Radiography With nanoDot Optically Stimulated Luminescence Dosimeter (OSLD)

**DOI:** 10.7759/cureus.34585

**Published:** 2023-02-03

**Authors:** Inayatullah Shah Sayed, Nurul Shuhada Roslan, Waliullah Shah Syed

**Affiliations:** 1 Department of Diagnostic Imaging and Radiotherapy, International Islamic University Malaysia, Kuantan Campus, Kuantan, MYS; 2 Department of Applied Sciences, Stanford International College, Mississauga, CAN

**Keywords:** exposure, bucky table, nanodot osld, dosimetry, backscattered radiation dose, radiation protection, radiography, esd

## Abstract

In radiography, inconsistencies in patients' measured entrance skin dose (ESD) exist. There is no published research on the bucky table induced backscattered radiation dose (BTI-BSD). Thus, we aimed to ascertain ESD, calculate the BTI-BSD in abdominal radiography with a nanoDot OSLD, and compare the ESD results with the published data.

A Kyoto Kagaku PBU-50 phantom (Kyoto, Japan) in an antero-posterior supine position was exposed, selecting a protocol used for abdominal radiography. The central ray of x-ray beam was pointed at the surface of abdomen at the navel, where a nanoDot dosimeter was placed to measure ESD. For the BTI-BSD, exit dose (ED) was determined by placing a second dosimeter on the exact opposite side (backside) of the phantom from the dosimeter used to determine (ESD) with and without bucky table at identical exposure parameters. The BTI-BSD was calculated as the difference between ED with and without bucky table. The ESD, ED, and BTI-BSD were measured in milligray (mGy).

ESD mean values with and without bucky table were 1.97 mGy and 1.84 mGy, whereas ED values were 0.062 mGy and 0.052 mGy, respectively. Results show 2-26% lower ESD values with nanoDot OSLD. The BTI-BSD mean value was found to be approximately 0.01 mGy.

A local dose reference level (LDRL) can be established using ESD data to safeguard patients from unnecessary radiation. In addition, to minimize the risk of BTI-BSD in patients in radiography, the search for the use or fabrication of a new, lower atomic number material for the bucky table is suggested.

## Introduction

Since the late 19th century, when x-rays, radium, and radioactivity were uncovered, radiologic imaging has undergone extraordinary scientific, clinical, and technological advancements that have continued to revolutionize medicine. X-rays are used to diagnose numerous disorders by visualizing the body's internal structures and have enhanced the effectiveness of medical practices. However, patients who are subjected to radiation are concerned, as x-rays have been linked to an increased risk of cancer [1,2]. Medical imaging is the primary source of ionizing radiation dosage for the general population [3]. Of the various medical imaging techniques, plain radiography of the abdomen has remained the first-line imaging method to assess and diagnose the disease if the patient has unexplained nausea, vomiting, or acute pain in the abdominal area or lower back. An abdominal x-ray examination can be performed to diagnose stones in the gallbladder, kidney, urinary bladder, intestinal blockages, perforation of the intestine, and constipation. In addition, x-ray imaging procedures are mandatory in urgent medical conditions even if the physician has reviewed the patient's medical history and laboratory tests [4].

Diagnostic images should contain sufficient details to allow for accurate medical decisions [5]. In many instances, high radiation dosages are often used to obtain higher-quality images; however, this increases patients' exposure to radiation. The potential loss of diagnostic data owing to a low radiation dosage must be evaluated against the increased risk of cancer associated with a higher radiation dose [6-9]. A rule of "as low as reasonably achievable" (ALARA) should be observed during radiological examinations to minimize these risks [10,11]. To prevent the escalation of health hazards, it is crucial to be aware of the total dose received by a patient during a radiologic procedure. Even though the radiation exposure from x-ray scans is modest, the increasing frequency of tests has sparked public concern. By publishing the patient dose report in diagnostic radiology in 2009, for instance, the National Radiological Protection Board (NRPB) of the United Kingdom (UK) contributed to increasing global awareness of measuring patient doses [12-14].

With the advancement of medical imaging technology, imaging quality has improved, resulting in an increase in the demand for radiological procedures year after year [15]. For this reason, it is crucial to monitor each patient's dose during a clinical x-ray examination to reduce the risk of cancer from radiation exposure and ensure that radiation protection practice is done properly [16-20]. In this regard, the entrance skin dose (ESD) and exit dose (ED) are crucial metrics used to evaluate the dose received by a patient during each medical x-ray imaging procedure. The ESD is the absorbed dose in air near the patient's body surface in the x-ray beam's center, including backscattered photons from the patient's body [21]. The patient’s dose is greatest at the surface of the patient’s body, and the deterministic effects normally occur on the skin [22]. The most common way to measure ESD and ED is to use dosimeters or mathematical tools to measure them directly on a patient or whole-body anthropomorphic phantom [23]. The ESD is also used to determine the diagnostic reference level (DRL) in diagnostic x-ray imaging. Furthermore, ESD is the most appropriate way to make sure that the clinical examination doses are within the DRL because this quantity is a key radiation protection criterion for benchmarking and comparing DRLs at the local, regional, and global levels [24]. The European Union has recognized the ESD to be observed as a DRL in radiology examinations to optimize patient dose. However, DRLs only act as guidelines and are not applied directly to individual patients or examinations. In addition, the ED is the factor that has been defined as the radiation dose received by the skin opposite an irradiated surface [25]. Renata and Gabriela used the exit air kerma as one of the patient dose estimation parameters in computed radiography (CR) [26]. 

In radiography, the backscattered radiation dose to the patient from the bucky table has never been considered. The backscatter radiation dose issue may not be considerable in posteroanterior (PA) erect abdomen radiography since there is no use of the bucky table. In addition, in the United Kingdom, the erect abdominal radiograph is practically extinct in clinical practice. Studies reveal that erect projection seldom impacts how a patient is managed [27]. Nonetheless, the radiation doses received by patients from the backscattered radiation induced by the bucky table could be smaller as compared to ESD. However, the linear no threshold (LNT) model sets no threshold dose for biological effects, such as the risk of cancer. LNT assumes that radiation can cause harm at any dose level, no matter how little, and that the accumulation of multiple very small exposures has the same probability of causing a stochastic health consequence as a single bigger exposure of equivalent dosage value [28]. The LNT model is still the most popular choice for estimating the likelihood of developing cancer in a population that has been exposed to radiation [29-31].

Literature reveals that there are inaccuracies in the patient dose measurements due to several factors, such as the use of different types of dosimeters, backscatter factors, tube output, tube voltage, filtration, collimation, focus-to-skin distance, and patients' size [10,23]. The most commonly used dosimeters are ionization chambers (ICs), dose area product (DAP) meters, and thermoluminescent dosimeters (TLD) [32]. In recent years, the optically stimulated luminescence dosimeter (OSLD) has become the preferred selection over other dosimeters, for example, the TLD in radiography [33,34]. Optically stimulated luminescence dosimeter OSLD is made of Al2O3:C (carbon-doped) by LANDAUER Inc. (Glenwood, IL). OSLDs are commonly used for wide energy ranges for low doses because they have potential dosimetric qualities like consistent sensitivity (still debatable), high precision and accuracy, fast readout, the ability to do repeated re-analyses, and dose accumulation [35]. The three steps that make up the foundation of OSLD's ionizing radiation detection process are as follows: the dosimeter is subjected to ionizing radiation; the radiation energy deposited there can cause excitations and ionizations; and is characterized by a metastable concentration of trapped electrons and holes. To retrieve the radiation dosage stored by the dosimeter, a light stimulus is applied. This is done by shining a certain wavelength of light (green) on the dosimeter, which moves the electrons to the conduction band and causes the electrons and holes to recombine back together. After that, a defect is made in the excited state, and the excited state returns to the ground state by giving off a photon (blue light), which is detected and measured by the OSLD reader [36].

The radiation dose for abdominal radiography is higher than for chest and extremity x-rays. This is because most of the abdominal organs are soft tissues with low electron densities, necessitating more milliampere-seconds (mAs) during imaging in order to more accurately evaluate them, and the kVp is within the photoelectric effect range [37-39]. The difference in densities between the structures, which is directly connected to their atomic numbers, explains why it takes fewer mAs to view structures like the bones [40-42]. Furthermore, exposure factors, such as kVp, mAs, and source-to-image distance (SID), are among the others that significantly affect the dose to patients. Additionally, the backscattered x-ray photons that are induced by the bucky table also contribute to the patient dose. As a result, it is critical to investigate the implications of these factors on patient dose and the proper implementation of the ALARA principle in radiography in general for the protection of patients from the adverse effects of ionizing radiation. Therefore, the objectives of this work were to ascertain ESD and ED values in abdominal radiography using nanoDot OSLD with and without bucky table by comparing the findings of this study with the other published data. Also, to estimate bucky table induced backscattered dose (BTI-BSD), which is caused by backscatter radiation induced by the bucky table, as BTI-BSD in radiography has not yet been studied.

## Materials and methods

A calibrated Siemens Multix Top x-ray system (Munich, Germany) was used. The whole-body phantom PBU-50, manufactured by Kyoto Kagaku (Kyoto, Japan), was exposed. The materials have radiation absorption and a Hounsfield number that is approximately equal to that of the human body. It has movable joints that allow for positioning in plain radiography and research. The 165 cm long, 50 kg phantom is built of human soft tissue and organ equivalents cast in urethane-base resin. The epoxy-based resin is used to make synthetic bones. Epoxy and urethane reinforced with carbon fiber are used in the joint attachments, while polycarbonate is used for the screws. Metal parts or liquids are not present in the body implants.

**Figure 1:**
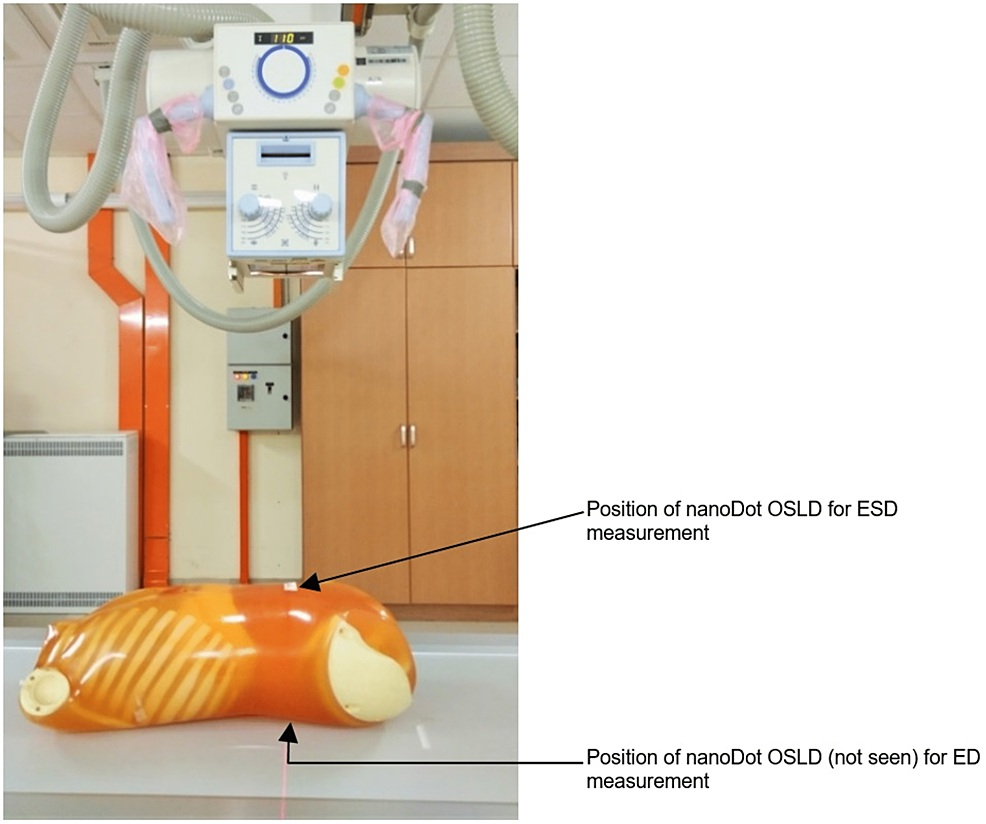
The phantom is shown with a bucky table. The image is owned by the authors of this study.

In this study, the abdominal area of the phantom was exposed, which included soft tissues, the liver, and the kidney. The exposures used were 70 kilovoltage peak (kVp) and 20 mAs, 75 kVp and 25 mAs, 81 kVp and 28 mAs. This range of exposures is employed in different hospitals in Malaysia for abdominal radiography. Furthermore, Thakur et al. assessed patient doses in the abdominal CR by selecting 81 kVp [43]. A large focal spot was selected to expose the abdominal area. The anti-scatter grid with a ratio of 8:1 was used. The phantom was placed on the bucky table in an antero-posterior (AP) supine posture as shown in Figure 1. The bucky table used is a fully floating tabletop fixed with a base made of a radiolucent material (composite) with a 0.7 mm aluminum equivalent x-ray absorption.

**Figure 2:**
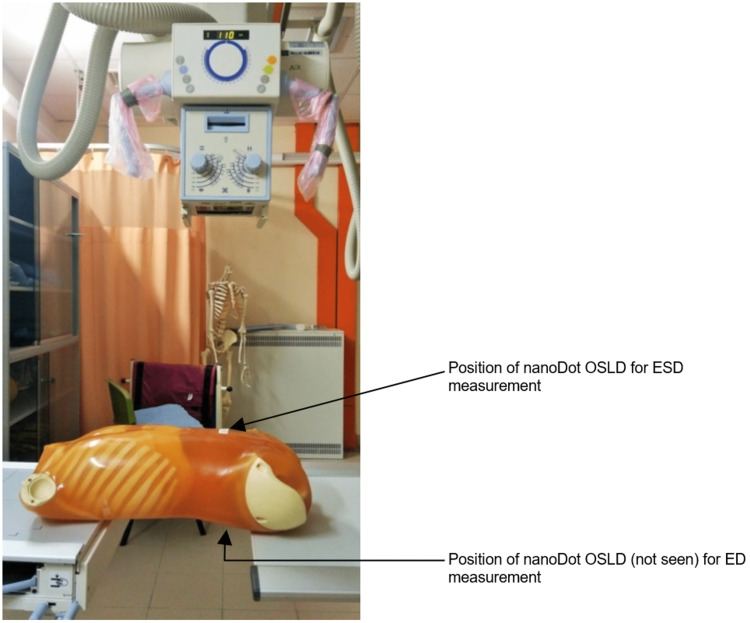
The phantom is shown without the bucky table. The image is owned by the authors of this study.

The source-to-image distance (SID) was fixed at 110 cm. According to the literature, keeping the kVp and mAs constant, and increasing the SID in abdominal examinations from 100 cm to 130 cm can optimize the examinations to maintain the patient dose as low as reasonably achievable [44,45]. The iliac crest level was identified as the location of the central ray, and the abdominal region was collimated adequately, including the lateral soft tissues. The technical parameters and details are shown in Table 1. Furthermore, Figures 1, 2 show the location of a nanoDot OSLD on the surface of the abdomen at the navel facing the central ray of the x-ray beam to measure the ESD. Also, both figures demonstrate how ED was measured by placing a nanoDot OSLD opposite the one used to measure the ESD.

**Table 1:** The technical parameters and details applied in this study.

Parameters	Details
Tube voltage (kVp)	70, 75 and 81
Tube current-exposure time (mAs)	20, 25 and 28
Source-to-image distance (cm)	110
Central ray	Surface of the abdomen at the navel
Focal spot	Large focal spot
Grid	8:1 ratio
Automatic exposure control (AEC)	Off

The dosimetry system by LANDAUER Inc. consists of nanoDot OSLDs, and a calibrated microStar reader unit by LANDAUER supplier was used. For the nanoDot OSLDs to be used again, they are optically bleached. This is a process where a light source is used to erase the dosimeter signals and make them about the same as the background signals. The dosimeter's sensitivity is then re-characterized based on its history. We performed a bleaching test on all nanoDOT OSLDs before exposing them to x-rays to rule out any residual radiation dosage from earlier studies. Also, each nanoDot was read with the microStar reader before it was exposed to radiation, and the background signal for each OSLD was recorded. This signal was then subtracted from the measured dose. The nanoDot OSLDs were sealed inside a pouch made of black polythene before being exposed or read.

Each exposure was repeated three times to get the average dose. After three exposures with the bucky table, the phantom was exposed at the same kVp and mAs without the bucky table to measure the ESD and ED (Figure 2). The phantom was exposed 18 times in total (with and without the bucky table).

The values of ESD and ED in mGy were directly recorded. A nanoDot OSLD was scanned and then placed in a microStar reader. The microStar system's sensitivity is 0.70. The SI unit for the radiation dose employed is mGy. The difference between ED data with and without bucky table was used to figure out the BTI-BSD using the equation; BTI-BSD = ED (with bucky table)-ED (without bucky table).

The percentage difference in ESD and ED at different exposures with and without bucky table was calculated by using the following equation: percentage difference = (V_1_-V_2_)/({V_1_+V_2_}/2)×100. Where V_1_ is the value of ESD or ED without bucky table and V_2_ is the value of ESD or ED with bucky table at different exposures [46]. The standard deviations in ESD and ED were calculated using the CalculatorSoup Online Calculators (Ashland, MA: CalculatorSoup, LLC) [46]. Furthermore, for the significance of results, t-test was performed at p<0.05.

## Results

ESD and ED with and without bucky table

**Figure 3:**
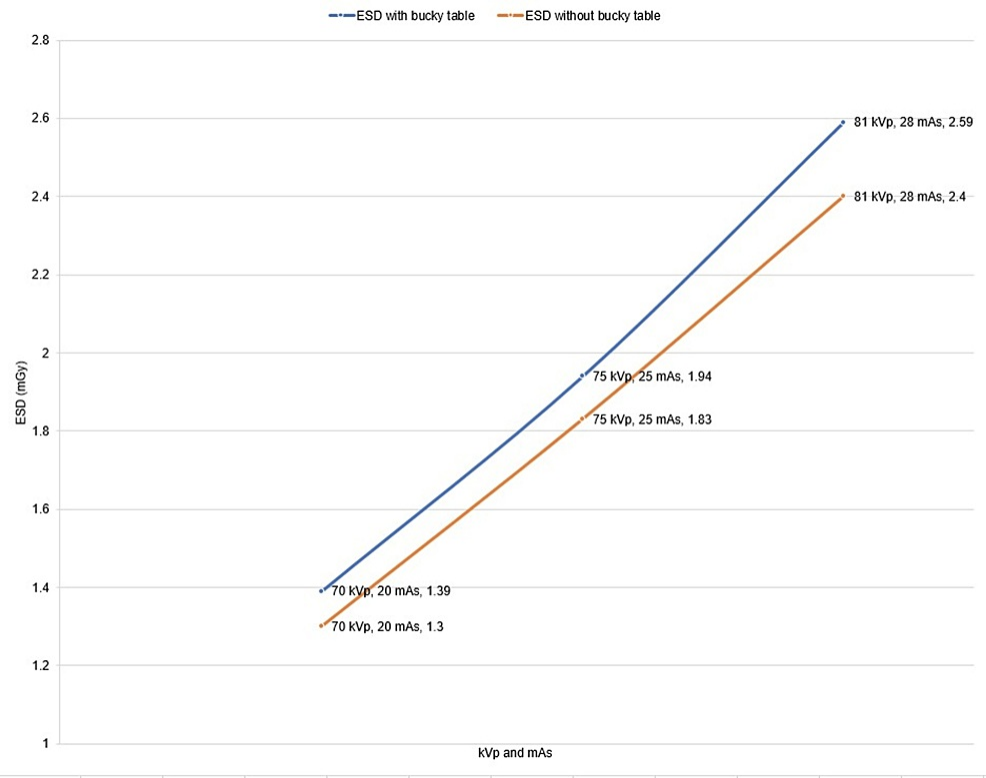
Shows the entrance skin dose with and without a bucky table. The image is owned by the authors of this study. ESD: entrance skin dose

Figure 3 shows the results of ESD measured by using the nanoDot OSLD with and without a bucky table. The ESD was obtained from the exposed OSLDs. The highest value of ESD is 2.59 mGy at 81 kVp with the bucky table. Generally, as the kVp and mAs increased, the ESD increased.

**Figure 4:**
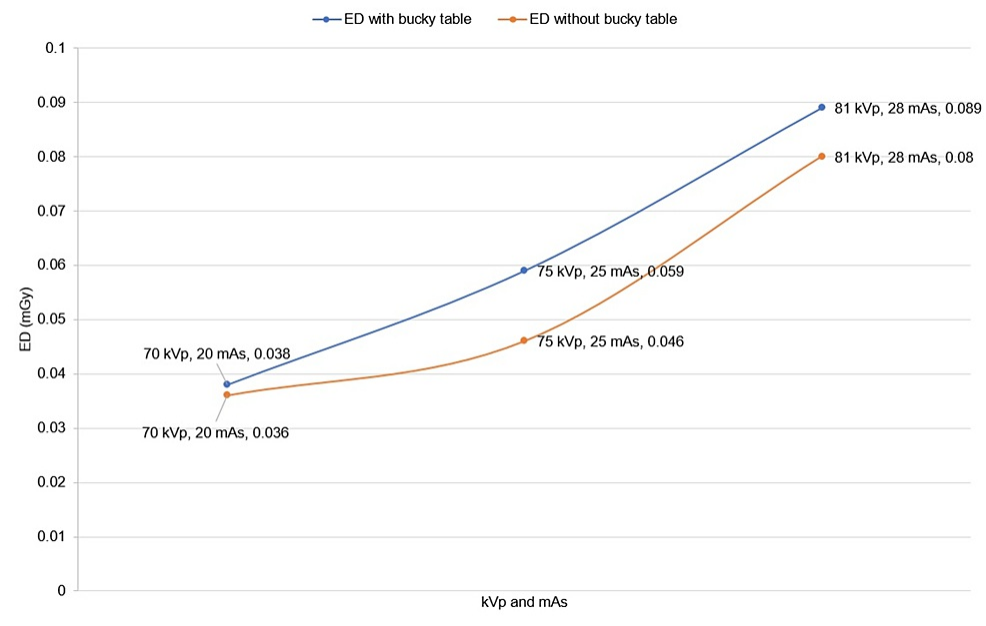
Shows the exit dose with and without a bucky table. The image is owned by the authors of this study. ED: exit dose

The ED values were obtained with and without the bucky table. Figure 4 presents the ED with the same kVp and mAs as selected in the case of ESD measurements. The highest value of ED obtained was 0.089 mGy with the bucky table. In addition, this shows the same trend, i.e., an increase in kVp and mAs caused an increase in ED.

Comparison of ESD with and without bucky table

Table 2 shows the percentage difference between ESD with and without bucky table. For each exposure, the ESD with a bucky table is higher than that without a bucky table. The percentage decrease in ESD without a bucky table compared to a bucky table for exposures of 70 kVp is found to be 6.6% and 75 kVp 5.8%. Furthermore, at 81 kVp, the decrease is 7.6%. On average, a 6.6% difference (increase) in ESD with bucky table relative to those without bucky table is recorded.

**Table 2:** Comparison of ESD with and without bucky table. ESD: entrance skin dose

kVp	mAs	ESD with bucky table (mGy)	ESD without bucky table (mGy)	Percentage difference (%)
70	20	1.39±0.026	1.30±0.024	6.6
75	25	1.94±0.040	1.83±0.039	5.8
81	28	2.59±0.027	2.40±0.023	7.6

Comparison of ED with and without bucky table

Table 3 summarizes the exit dose with and without the bucky table. The exit dose without the bucky table is lower than with the bucky table. The percentage difference (decrease) in ED without bucky table compared to a bucky table ranges from 5.4% to 24.7%. In addition, a 5.4% difference at 70 kVp in ED with bucky table compared to without bucky table is obtained. Other exposures of 75 kVp and 81 kVp resulted in a 24.7% and 17% decrease in ED without bucky table compared to those with bucky table, respectively. The mean difference (decrease) in ED without the bucky table compared to with the bucky table is found to be 15.7%.

**Table 3:** Comparison of exit dose with and without bucky table.

kVp	mAs	Exit dose with bucky table (mGy)	Exit dose without bucky table (mGy)	Percentage difference (%)
70	20	0.038±0.008	0.036±0.007	5.40
75	25	0.059±0.012	0.046±0.009	24.76
81	28	0.089±0.018	0.075±0.015	17.07

Comparison of ESD with other published data

Table 4 presents the comparison of the ESD values with the bucky table at different kVp and mAs obtained from this study with the other published data [2,10,15,47]. The ESD value of this study is lower compared to other published data.

**Table 4:** Comparison of the ESD of our study (without the bucky table) with other published data. ESD: entrance skin dose

Examination		This study	Aliasgharzadeh et al. (2015) [2]		Rasuli et al. (2017) [10]	Taha et al. (2015) [15]		Moey et al. (2017) [47]
Abdomen	kVp	75.3		73	67.1		83	74.9
	mAs	24.3		24	37.2		12.5	40.38
	Dose (mGy)	1.97		2.01	2.32		2.10	2.57
Percentage difference		2-26		2	16		6	26

Bucky table induced backscattered radiation dose results

The BTI-BSD for 70 kVp, 75 kVp, and 81 kVp, was found to be 0.002, 0.013, and 0.014 mGy, respectively. The highest BTI-BSD was measured at 81 kVp.

## Discussion

To determine the risk associated with a radiographic examination, it is necessary to know the dosage absorbed by each organ and the associated risk. The entrance surface dose (ESD) determines the dosage delivered to a patient's organs and tissues during radiography procedures. In addition, without the bucky table, abdominal imaging cannot be done in practice, which induces backscatter radiation. There is no available literature on BTI-BSD that patients get during a clinical x-ray examination. The estimated ESD of x-rays in medical examinations is between 0.2 and 7.8 mGy. That is far larger than the nanoDot's lower detection limit of 5x10^-4^ mGy. Even at 3 μGy, the reliability of OSLD was reported to be ±15%. Thus, the nanoDot OSLD is thought to be a good radiation dosimeter for monitoring low doses of radiation [48].

This research focused on measuring the ESD, ED, and BTI-BSD in abdominal radiography by irradiating a phantom with nanoDot OSLD. The findings of the study demonstrate that ESD and ED increased as the tube voltage and mAs increased. Higher kVp and mAs enhance x-ray photon intensity, allowing more x-ray photons to hit nanoDot OSLD [24]. When the x-ray beam travels through the patient's body, there are three possibilities that can occur; (i) the x-ray photons pass through the body without any interaction, (ii) some of the x-ray photons can interact via photoelectric effect and transfer all of the energy into the patient’s body, and (iii) after the interaction, the x-ray photons lose part of their energy and scatter dominantly through Compton interaction. Scattered x-ray photons can travel in any direction (forward scatter and backscatter). Furthermore, backscattered x-ray photons can reflect back at different angles. Backscattered radiation can be reflected from hard surfaces such as bucky table. In this case, the patient can receive an additional radiation dose because the distance between patient and the bucky table is short. Therefore, a suitable material for the bucky table is important to reduce the backscattered radiation dose.

**Figure 5:**
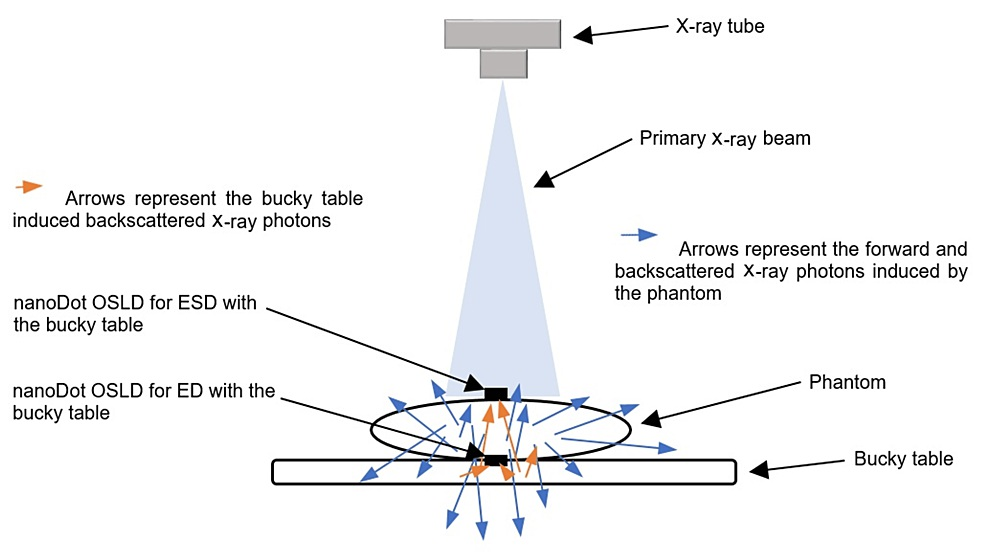
The forward and backscattered radiation induced by the phantom and bucky table. The image is owned by the authors of this study.

**Figure 6:**
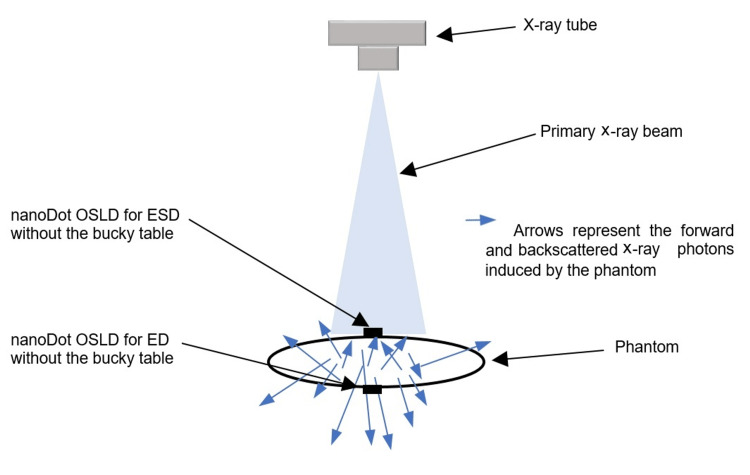
The forward and backscattered radiation induced by the phantom. The image is owned by the authors of this study.

The ESD and ED are higher with the bucky table than without it. This is because the bucky table contributed to the increase in the dose due to the generation of backscattered x-ray photons. Therefore, more backscattered radiation from the bucky table reached nanoDot OSLD, which was used for the estimation of exit dose fixed at the phantom's back as compared to the nanoDot OSLD used to determine ESD (Figures 5, 6). Further, this can be confirmed by higher ED values with the bucky table as compared to without the bucky table. It is worth mentioning that nanoDot OSLD positioned for ESD estimation recorded on average a 6.6% increase in dose with bucky table compared to without bucky table. The lesser increase in ESD compared to ED could be due to a lower number of bucky table induced backscattered radiations reaching the nanoDot OSLD for ESD versus the nanoDot OSLD used for ED. It means that 6.6% BTI-BSD contributed to the ESD value, hence ESD should be corrected for. Whereas a significant increase of 15.7% (p<0.05) on average in ED with bucky table compared to without bucky table was recorded.

In this study, the kVp and mAs used were a little bit different from those used in other studies [2,15,10,47]. The ESD obtained from this study revealed a 2-26% decrease in ESD values compared with the published data [2,15,10,47]. This study utilized the Kyoto Kagaku phantom while the other published data obtained the ESD values from real patients. The anatomical structures between these two subjects are different as humans have more organs and soft tissues in the abdominal region. As a result of this property, the x-ray beam's penetrability, absorption, and scattering of photons may be modified. Other than that, the x-ray tubes installed were different from one institution to another. Each x-ray system has different characteristics, such as the generator. The x-ray generator allows the adjustment of electrical quantities that determine the production of x-rays and affect the radiation output. Several other factors that need to be included are tube output, tube voltage, tube loading, focus-to-skin distance, and backscatter factors [18,19]. In addition, there is a possibility that different dosimeters cause a difference in ESD. This study utilized the nanoDot OSLDs while the other published data obtained the ESD values using different dosimeters, e.g., dose area product (DAP). Endo et al., Al-Senan and Hatab, and Lim et al. were able to effectively evaluate how OSLDs (nanoDots) responded to x-rays in the diagnostic energy range [34,49,50]. With an average signal loss of less than 2.5% over the course of three repeated readouts, it is found that the nanoDots demonstrate high linearity and repeatability in the chosen radiation quality [51]. Studies done by Endo et al., Al-Senan and Hatab, and Lim et al. have also looked at the dosimetric properties of OSLDs and found that they work well in the diagnostic energy range of 40-140 kVp [34,49,50]. Also, the difference in ESD may be caused by several things, such as differences in patient weight and exposure factors [52,53].

Furthermore, numerous studies have computed ESD using specialized formulas and various radiation parameters, such as x-ray device output [54-56]. So, the different ways of figuring out ESD could be one of the reasons why the measured dosage values aren't the same. This is in line with what Vassileva et al. and Gholami et al. found, and it may be because there aren't enough qualified health physicists to teach how to choose the best kVp, mAs, film-focus distance (FFD), and calibration method [57,58].

Researchers from several institutions have noted significant variations in the radiation doses that patients underwent during x-ray procedures. Thus, researchers studying patient doses around the world have recently become interested in these variations in patient dosimetric values seen in many different countries. The growing understanding of the danger posed by low ionizing radiation doses has highlighted the urgent need for dose assessment due to radiological x-ray examinations [52,53]. The doses measured in different countries must be compared to the exact standards set by the National Radiological Protection Board (NRPB), the International Atomic Energy Agency (IAEA), and the Commission of the European Communities (CEC) [6]. The European Commission (EC) recommends that the entrance surface dosage be monitored as the primary radiographic dose measure. The European Union has set a diagnostic reference level (DRL) for this amount so that the dose to the patient may be optimized without losing the benefits of a diagnostic x-ray examination. Before trying to figure out if diagnostic x-rays could hurt a patient, it is important to know how much ionizing radiation is absorbed by vital organs and tissues [59].

The International Commission on Radiological Protection (ICRP) eventually recommended utilizing the metric of normative values for patient radiation exposure from a given procedure, which was first used in the United Kingdom in 1990 [60]. For certain imaging methods, these normative measurements, called diagnostic reference levels (DRLs), are equal to the 75th percentile of the recorded dose values [61]. Furthermore, several agencies and authorities have established diagnostic reference levels (DRLs) to analyze and report the levels of ionizing radiation exposure and its effects. A United Nations Scientific Committee on the Effects of Atomic Radiation (UNSCEAR) report from 2000 says that uncontrolled use of ionizing radiation may become carcinogenic, which means that it could cause cancer [62,63]. Additionally, the ICRP stresses that DRLs shouldn't be used for specific patients. If the protocol remains constant, the average data from several facilities should be compared to the DRL standard to establish meaningful comparisons. However, radiology departments in one city could employ lower doses than international DRLs. The dosage that patients receive can be decreased with the use of the DRL formulation [37,64]. The Malaysian government has conducted the nationwide dosage survey twice, from 1993 to 1995 and again from 2007 to 2009. To guarantee that the use of ionizing radiation is monitored and optimized, all centers use the national dose survey conducted by the Ministry of Health (MOH) as a guide [65].

Ionizing radiation is important and helps with disease diagnosis, but sometimes more dose exposure is given than is required. In general, an optimized dose in medical imaging is a balance between the image quality and radiation dose that is enough for the scope of the inquiry to achieve the specific clinical objectives [14]. In May 2013, the Malaysian Ministry of Health released its Medical Radiation Exposure Report, which set its DRL for radiological operations. The construction of local facility DRLs can help hospitals partially meet the rules' needs for the assessment and evaluation of patient dosages for medical radiological examinations. Local facility DRLs should be created and then contrasted with national DRL values. To make sure that best practices and expected results are met, an investigation must be conducted if local facility DRLs often exceed national DRL values or are much lower than national DRL values. When problems are found, a risk analysis or inquiry must be conducted to determine potential reasons. Corrective measures should be put in place to reduce the hazards to patients. Therefore, the ESD values we obtained in this work might help establish LDRLs and improve current practices for patient radiation protection.

Radiation causes deterministic and stochastic cellular damage when there is insufficient time for repair. The cause of stochastic consequences, such as cancer and hereditary impacts, is a mutation or other long-lasting alteration in which the cell retains viability. DNA base changes can occur with a small dose of x-rays. Accordingly, the probability of a stochastic effect increases with dosage (likely without a threshold, based on molecular carcinogenesis information), but dose has nothing to do with how severe the effect is [66]. Leading international and national radiation expert groups concluded in 2005-2008 that biological and biophysical facts support a linear no threshold cancer risk model. For example, the response to dose at low levels is usually linear, and there is no evidence of a threshold [18,67]. Thus, BTI-BSD should not be ignored, even though this study found a very low dose (0.01 mGy).

There are no similar studies that have looked at the BTI-BSD in abdominal radiography. As a result, the findings of this work cannot be compared. The calculated result for BTI-BSD shows a low dose of 0.01 mGy. This illustrates that the radiation doses received by patients from BTI-BSD were low. However, as mentioned earlier, the LNT model sets no threshold dose that could result in biological effects such as an increased risk of cancer, which means that even a low level of radiation dose may have health implications. In the context of this study, high weight is given to the LNT model in terms of risk assessment due to its wide acceptability as supported by researchers [29-31]; therefore, even a lower value of BTI-BSD can increase the risk of health complications. Thus, dose assessment is required to safeguard patients from radiation and provide the lowest dose during clinical tests in radiology since x-rays are an ionizing radiation source and their usage is not entirely risk-free.

## Conclusions

This study is the first to assess BTI-BSD. The BTI-BSD increased with the increase of kVp. Compared to published data, the result shows that ESD is 2-26% lower. The measured BTI-BSD is low (0.01 mGy), while the LNT model does not set a threshold dose for biological effects of ionizing radiation, such as the risk of cancer. Although the BTI-BSD values recorded were low, even the smallest amount of radiation dose is harmful. As a result, it is important to protect patients from excess radiation doses caused by bucky table induced backscattered x-ray photons. This study's ESD values can be used to establish LDRL and improve radiation protection practices for patients. Furthermore, a new material with a low atomic number for bucky tables could be used or discovered to reduce the BTI-BSD risk for patients undergoing radiographic procedures.
